# Utilizing age-friendly cities and communities to support access to vaccination, vision, hearing and oral healthcare

**DOI:** 10.3389/fragi.2026.1805946

**Published:** 2026-04-29

**Authors:** Cera Cruise, Katrina Bouzanis, Anna Sangster, Sarah Hyeon-A. Kim, Jane Barratt

**Affiliations:** 1 International Federation on Ageing, Toronto, ON, Canada; 2 Department of Geography and Planning, University of Toronto, Toronto, ON, Canada

**Keywords:** age-friendly, hearing care, immunization, oral health, vision care

## Abstract

Population aging is a global phenomenon, resulting in increasing numbers of older people. As populations age, the prevalence of noncommunicable diseases and sensory loss also increases. These challenges create an imperative to maintain health across the life course, to reap the benefits of longer lives and reduce the burden on health and social systems. Functional ability is shaped by the individual and the environment in which people live. The World Health Organization’s Age-friendly Cities Framework defines an age-friendly city as one that adapts its structures and services to be accessible to and inclusive of older people with diverse needs and capacities and is guided by eight domains, including community support and health services. Despite the linkage between age-friendly environments and health, the Age-Friendly Cities Framework can be better harnessed to support promoting access to health services which contribute to function but are not always central to health planning: immunization, and vision, hearing and oral care. Examination of 59 strategy and action plans submitted by members of the Global Network of Age-Friendly Cities and Communities (GNAFCC) indicates that only 18 out of 59 cities and communities included health actions to support vaccination, hearing, vision and oral health in their age-friendly plans. This article explores the importance of these interventions to healthy aging, the extent to which age-friendly cities and communities support access to these health services within their plans and how age-friendly planning may be utilized as a tool to enhance comprehensive access to immunization, vision, hearing and oral care.

## 1 Introduction

By 2030, approximately one in six individuals globally will be aged 60 years or older, amounting to an estimated 1.4 billion people. Although life expectancy continues to increase, the number of years lived in good health has not kept pace. As individuals age, the ability to maintain functional abilities and intrinsic capacity evolves and is significantly influenced by the physical and social environments in which they live. Functional ability is shaped by the interaction between personal capacities and environmental conditions (World Health Organization, 2015; World Health Organization, 2020). Ideally, this interaction enables older people to meet basic needs, pursue opportunities for learning and decision-making, maintain mobility, establish and nurture relationships, and contribute to society (World Health Organization, 2020).

In 2015, the World Health Organization (WHO) introduced a public health framework for optimizing healthy aging across the life course, which stipulates that environments, which include the availability of health services and long-term care, are central to functional ability by promoting capacity-enhancing behaviour and/or removing barriers to participation and compensating for loss of capacity (World Health Organization, 2015). To support the creation of environments that enable healthy ageing, the World Health Organization developed its Age-friendly Cities Framework (World Health Organization, 2007). The interrelationship between functional ability and environmental conditions is central to this framework, which defines an age-friendly city as one that adapts its structures and services to be accessible to and inclusive of older people with diverse needs and capacities (World Health Organization, 2007). The framework identifies eight interconnected domains that support age-friendly development: outdoor spaces, transport and mobility, housing, social participation, social inclusion and non-discrimination, civic engagement and employment, communication and information, and community support and health services (World Health Organization, 2007).

According to this framework, an age-friendly city or community importantly includes access to comprehensive and appropriate health services (World Health Organization, 2007). Despite this clear linkage, there appears to be a lack of connection between age-friendly planning and ensuring the availability of certain health services to support prevention of disease, diagnosis, and access to treatment, aids or rehabilitation.

Major contributors to functional ability particularly prevention of disease through vaccination, and access to health services not often included in universal health coverage, such as hearing, vision, and oral care services, within age-friendly action plans is not well understood and illustrates an important opportunity for more comprehensive age-friendly programming that responds to growing needs across these areas. Vaccine-preventable diseases, hearing and vision loss, and poor oral health greatly impact health and quality of life of older people, contribute to health system burden and have broad socioeconomic impact (Janto et al., 2022; El Banhawi et al., 2024; de la Fuente-Núñez et al., 2023; World Health Organization, 2021; Privor-Dumm et al., 2021).

An exploration of age-friendly cities and community action plans was conducted to understand whether vaccination, hearing, vision and oral care services are including in age-friendly planning. This exploration surfaced initiatives and highlighted gaps in the intersection between immunization, hearing, vision and oral care services and age-friendly planning and the need to bridge sectors, align agendas and understand opportunities to enhance the creation of enabling environments by promoting access to health services central to healthy aging.

## 2 The WHO Global Network for Age-Friendly Cities and Communities

The Global Network for Age-friendly Cities and Communities (GNAFCC) was established in 2010 to connect, support, and inspire cities and communities around the world to become more age-friendly (World Health Organization, 2018). Over 1,500 cities and communities from five of the six WHO regions (Americas, South-East Asia, Europe, Eastern Mediterranean, and Western Pacific) are members of the GNAFCC, demonstrating a potential for large scale action to respond to issues impacting older adults.

The Age-friendly Cities Framework directly informs the GNAFCC. Any local or sub-national level of government in WHO Member States can apply to be a member of the GNAFCC, and all members share and update the age-friendly strategy and action plans via the GNAFCC portal, providing a rich source of publicly available data on Age-friendly World, the WHO’s web platform which hosts the GNAFCC, the Global Database of Age-friendly Practices, and other age-friendly resources shared by GNAFCC members (Age Friendly World, 2025).

Despite community support and health services being one of the eight domains central to age-friendly planning, there is a paucity of efforts to support key public health actions related to vaccination, and vision, hearing and oral care. For example, an examination of 59 strategy and action plans submitted by members admitted to the GNAFCC between 1 January 2020 and 28 September 2023 found that vaccination, hearing, vision and oral health actions were rarely noted in plans. A total of 18 cities and communities in seven countries of the 59 analyzed cities and communities included health actions to support vaccination, vision, hearing and/or oral care within their age-friendly strategy and action plans.

This finding demonstrates that there is a need to make the case for the importance of including health promotion efforts and supporting access to health services, which greatly support functional ability, into age-friendly planning.

## 3 Vaccination as a component of age-friendly cities and communities

As the population ages globally along with rising rates of noncommunicable diseases, there is a growing population of adults at-risk of vaccine preventable diseases (VPDs), such as influenza, pneumococcal pneumonia, COVID-19, respiratory syncytial virus, and shingles. For older persons and those with underlying chronic conditions, VPDs result in significant morbidity, mortality, hospitalization and lasting declines in function (Doherty et al., 2022; Bell et al., 2022; Ackerson et al., 2019; Jiang et al., 2025; Talbird et al., 2020). Beyond the individual, VPDs have a significant public health impact, burdening health and social care systems and healthcare professionals. There is an increasing recognition of the economic and social benefits of vaccines in the development and assessment of vaccine programs, potentially realizing a greater benefit to society and raising support for wider implementation (El Banhawi et al., 2024).

Despite benefits to individuals, communities and health systems broadly, there continues to be gaps in vaccine coverage and uptake (Doherty et al., 2019; de Gomensoro et al., 2018), and a lack of connection between immunization and healthy aging strategies, including age-friendly programming.

In a review of AFCC action plans and strategies, only nine out of 59 (15%) included vaccination as an item within their age-friendly strategy and action plan. This is a greatly missed opportunity, given the known role of community in promoting vaccination and recognition of social norms and processes as a key driver of the motivation to receive vaccination (Xie et al., 2024; World H ealth Organization, 2022). AFCC can also be a clear enabler to ensuring vaccines are available to older adults and promoting clear and accessible information on immunization.

Cities and communities that included vaccination within age-friendly strategy and action plans varied in their approaches to supporting older adults. Key actions included the inclusion of adult immunizations to the national immunization program, communication efforts to increase awareness of vaccination, and supporting access and administration (Table 1). These examples demonstrate practices that may be replicated and expanded upon in other jurisdictions.

**TABLE 1:** Description of actions noted by Global Network for Age-friendly Cities and Communities (GNAFCC) members addressing immunization, and hearing, vision and oral care.

Health service	Country	GNAFCC member	Description of actions
Immunization	Republic of Korea	Jung-gu	Supports access to pneumococcal pneumonia, influenza vaccination (Age-Friendly World, 2026a)
	Republic of Korea	Seogu	Supports access to pneumococcal pneumonia, influenza vaccination. (Age-Friendly World, 2026b)
	Republic of Korea	City of Jinju	Supports access to pneumococcal pneumonia, influenza vaccination. (Age-Friendly World, 2026c)
	Republic of Korea	Namhae-gun	Supports access to shingles vaccination. (Age-Friendly World, 2026d)
	Republic of Korea	Uiryeong-gun	Supports access to pneumococcal pneumonia vaccination. (Age-Friendly World, 2026e)
	Republic of Korea	Wanju-Gun	Supports access to COVID-19, pneumococcal pneumonia, influenza, and typhoid. (Age-Friendly World, 2026f)
	Canada	Grey County	Proposed using its Community Paramedicine Home Visit Program to assist in the administration of influenza vaccines to older adults, vulnerable residents, and those who provide healthcare to older adults. Partners identified as being potential supporters of this effort included a local integrated health coalition, a community health centre, the regional public health authority, and the municipally run-long term care provider. (Age-Friendly World, 2026g)
	Canada	Town of Lincoln	Creation of dedicated space on its municipal website sharing where residents could receive COVID-19 immunization. (Age-Friendly World, 2026h)
	Singapore	Singapore	Included general support for immunization as an age-friendly activity within its strategy and action plans but did not outline specific actions. Providing older adults with a health journal to include vaccination details was noted as a strategy to promote vaccine awareness and health. (Age-Friendly World, 2026i)
Ear and Hearing Care	Republic of Korea	City of Jinju	Provides hearing aids for low-income older adults. (Age-Friendly World, 2026c)
	New Zealand	Tāmaki Makaurau/Auckland	Completed housing development with adapted design for deaf individuals. (Age-Friendly World, 2026j)
	Israel	Herzliya	Healthy City program provides hearing testing for older adults using volunteers. (Age-Friendly World, 2026k)
	Singapore	Singapore	Recommends to their citizens to have an assessment for hearing loss, a way to raise awareness of the importance of hearing health. (Age-Friendly World, 2026i)
	Canada	Regional Municipality of Durham	Installed digital signs in buses to assist hearing impaired users. (Age-Friendly World, 2026l)
Vision Care	Wales, United Kingdom	Flintshire	Facilitates older adults being referred to an optometrist as a part of their Falls Prevention Service. (Age-Friendly World, 2026m)
	Republic of Korea	Namhae-gun	Supports vision correction services for low-income older adults. (Age-Friendly World, 2026d)
	Republic of Korea	Uiryeong-gun	Supports the costs of eye exams and surgery for low-income older adults. (Age-Friendly World, 2026e)
	Republic of Korea	Changwon City	Supports medical expenses of low-income and older adult residents for vision correction. (Age-Friendly World, 2026n)
	Canada	Grey County	Promotes information on services available to assist in the purchase of glasses. (Age-Friendly World, 2026g)
​	Canada	Regional Municipality of Durham	Recommends a future action of advocating for affordable and accessible vision care. (Age-Friendly World, 2026l)
	New Zealand	Tāmaki Makaurau/Auckland	Completed housing development with adapted design for visually impaired individuals. (Age-Friendly World, 2026j)
	Israel	Herzliya	Provides vision testing for older adults using volunteers. (Age-Friendly World, 2026k)
	Singapore	Singapore	Suggests providing a screening package to screen for vision loss. (Age-Friendly World, 2026i)
Oral Care	Northern Ireland, United Kingdom	Armagh City, Banbridge & Craigavon Borough Council	Identified environmental barriers to accessing local services and outlined commitment to provide service providers, such as dentists, a method to assess the extent to which their establishment was age-friendly. (Age-Friendly World, 2026o)
	Republic of Korea	Seogu	Supports oral prosthetic provision for people with disabilities and older adults. (Age-Friendly World, 2026b)
	Republic of Korea	City of Jinju	Provides dentures and implants for low-income older adults. (Age-Friendly World, 2026c)
	Republic of Korea	Namhae-gun	Provides dentures and implants for older adults. (Age-Friendly World, 2026d)
	Republic of Korea	Uiryeong-gun	Support for self-payment of dentures and implants for low-income older adults. (Age-Friendly World, 2026e)
	Republic of Korea	The City of Gwangju	Support of oral examinations for older adults. (Age-Friendly World, 2026p)
	Republic of Korea	Wanju-Gun	Assists in the co-payment of denture costs for adults 65 years of age and older who receive basic living medical benefits and secondary health insurance. (Age-Friendly World, 2026f)
	Canada	Nackawic	Proposes exploring the possibility of having consistent dental hygienist access in the community. (Age-Friendly World, 2026q)
	Canada	Grey County	Promotes information on services available to assist in coverage for oral healthcare. (Age-Friendly World, 2026g)
	Canada	Regional Municipality of Durham	Recommends a future action of advocating for affordable and accessible oral healthcare. (Age-Friendly World, 2026l)
	Türkiye	Bornova	Provide access to a dentist for specific at-risk groups. (Age-Friendly World, 2026r)

## 4 Hearing and vision health services as a component of age-friendly cities and communities

As reported by the WHO’s World Report on Hearing, globally, more than 1.5 billion people experience some degree of hearing loss, which translates to about 1 in 5 people, worldwide (World Health Organization, 2021). By 2050, this number is anticipated to grow by 60%, to 2.5 billion, which means that 1 in 4 people will experience hearing complications (World Health Organization, 2021). There is a known association between aging and hearing loss, with prevalence increasing after the age of 50 (Haile et al., 2021; Shen et al., 2025). The WHO finds that 42% of individuals with hearing loss are over 60 years old (World Health Organization, 2021).

The rise of hearing complications and the rapidly aging global population will significantly increase the incidence of those with hearing loss and therein loss of function. Hearing loss has a significant effect on the functional ability and wellbeing of older people. An individual with hearing loss may experience changes in balance, which in turn affects mobility and physical function, resulting in increased frailty, falls, and higher hospitalization rates (World Health Organization, 2021; Tian et al., 2021). Hearing loss also impacts the wellbeing of older adults, resulting in limitations in the ability to communicate with others, social isolation, depression, and often, increased dependence (Ciorba et al., 2012). Additionally emerging research finds that hearing loss might be a contributing factor to dementia risk over time, and that treating hearing loss may lower dementia risk (Lin et al., 2011; Livingston et al., 2020).

Similar to hearing loss, vision loss is also highly associated with aging. In fact, older adults experience the highest prevalence of preventable blindness (Bourne et al., 2021). Vision health greatly impacts functional ability and is instrumental to advancing healthy aging, by enabling people to be mobile and to interact safely with their peers, family, and their environment (de la Fuente-Núñez et al., 2023). Vision facilitates participation in everyday life, such as working, socializing, learning, and doing things that we enjoy (World Health Organization, 2019). Studies have found that visual impairment can limit the activities of older adults, which in turn results in limited independence, social isolation, loneliness, and depression (Brown and Barrett, 2011; Evans et al., 2007; Heesterbeek et al., 2017). Further research has shown that vision impairment is closely related to cognitive decline and the ability to manage other health conditions (Court et al., 2014; Varadaraj et al., 2021; Cao et al., 2021).

Given the large and growing prevalence of vision and hearing loss which affects various facets of living, health and wellbeing, environments can significantly support increasing functional ability by supporting awareness and education, access to ear and hearing care and vision health services, including testing and screening for hearing and vision loss, access to treatments (e.g., anti-VEGF injections, steroids, and other medications, surgical interventions), delivery of aids and assistive technology (e.g., hearing aids, corrective lenses), and rehabilitation, and supporting infrastructure design which maximizes accessibility for those with hearing and/or vision loss.

In reviewing age-friendly strategies and action plans of GNAFCC members, five cities and communities (8%) in five countries included hearing health within their age-friendly strategy and action plans, with actions aligned with the age-friendly domains of communication and information, housing, community support and health services, and transportation. Nine cities and communities (15%) in six countries included vision as an item in their age-friendly strategy and action plans, under the domains of community support and health services or housing (Table 1). The strategies outline interventions that may be prioritized to advance access to vision and ear and hearing care services and accessibility for those with vision and hearing impairment.

## 5 Oral health services as a component of age-friendly cities and communities

Oral diseases are highly prevalent in older people (Bernabe et al., 2020). Across the life course, factors such as diet, oral hygiene, fluoride exposure, smoking and access to dental care may impact oral health in later life (Aida et al., 2022). Additionally, aging is associated with physiological conditions and risk factors, such as the presence of chronic conditions and increased use of medications, that impact the oral health of older adults (Griffin et al., 2012). Aging as well as many chronic conditions are associated with a decrease in salivation, which may lead to chewing and swallowing difficulties, greater incidence of dental caries and periodontitis (Poudel et al., 2024), a disease of the tissue surrounding the tooth structure (Mehrotra and Singh, 2023). These conditions may lead to tooth loss and deterioration in general health, for example, affecting the ability to eat and receive proper nutrition, leading to frailty, difficulty in managing other chronic conditions, or negatively affect self-esteem due to changes in appearance (Poudel et al., 2024).

Certain chronic conditions may make it more difficult to manage oral health, however, declines in oral health may also lead to more systemic disease. For example, there is evidence that periodontal disease is a risk factor for cardiovascular disease, stroke and type 2 diabetes due to chronic inflammation associated with the disease (Kim and Amar, 2006).

Older adults often face significant barriers to accessing oral care services. Dental care is not often included in public health insurance programs, resulting in high out of pocket costs to receive care, and there have been calls to include dental care as part of universal health coverage (Bernabe et al., 2020). Additionally, there is oftentimes a lack of education on proper oral hygiene or an inability to access primary dental care. Access to oral care is an often-overlooked aspect of health promotion and prevention which may be supported in the creation of AFCC.

Actions to improve oral health were evident in about 19% of GNAFCC members studied, with examples demonstrated in four countries. All actions were captured in the community support and health services age-friendly domain. Actions included raising awareness of available services, improving access to oral health examinations and improving access to dentures and implants (Table 1).

## 6 Discussion

As supported by the Age-Friendly Cities framework, there is a clear intersection between the delivery of health services and the creation of age-friendly environments, yet these agendas oftentimes do not intersect and strategies to address health and social challenges remained siloed. While most age-friendly strategies acknowledge the role of health, few translate that recognition into concrete actions targeting vaccination, hearing, vision, and oral health. The age-friendly agenda can be leveraged across health disciplines to support access to care, yet there is failure from those involved in age-friendly planning and health sectors to collaborate to develop shared solutions which enhance policy and practice to improve health outcomes.

While there is often attention to infrastructure, such as housing, public spaces and transportation, AFCC can be key champions in ensuring in access to health education and services within the community. For example, Canada is a leader in age-friendly planning; the Canadian age-friendly communities’ program includes communities in at least 11 provinces and territories (Public Health Agency of Canada, 2025). The Public Health Agency of Canada (PHAC) has an overarching convening power every month in the form of a reference group that exchanges knowledge, opportunities, and challenges. Only two cities and communities in Canada were found to have interventions associated with vaccination. While this does not mean that Canadian cities and communities are not engaged more extensively in raising awareness and promoting vaccination, it highlights the underutilization of the age-friendly framework as a powerful vehicle to support these efforts. As part of the mandate of PHAC to promote age-friendly environments to improve older persons’ functional abilities, age-friendly frameworks could be leveraged more effectively to promote the value of adult vaccination.

Beyond the delivery of healthcare services, age-friendly planning may support efforts to prevent disease, such as through championing immunization, prevention of hearing loss by limiting exposure to noisy environments or promoting access to regular dental check-ups (World Health Organization, 2021; Patel and Gallagher, 2024). Additionally, AFCCs are important to support increased social participation for those affected by health conditions discussed above. For example, those with hearing and/or vision loss may benefit from universal building design, which aims to ensure that spaces and living environments are usable by all people without the need for adaptation (Centre for Excellence in Universal Design, 2025; Patil et al., 2025). For those with hearing loss, attention to ‘soundscapes’ in urban design is important, which considers the sound environment in combination with the human experience and behavioural response to it, rather than the noise level of the setting alone (World Health Organization, 2021).

The interventions currently implemented by GNAFCC members, such as increasing education and awareness of health services, supporting access to services through financial coverage, or connecting to healthcare providers or aids, or improving accessibility of environments for those with loss of function, provide a starting point for good practice and ways in which AFCC strategies and actions may support access to health interventions and health promotion in key areas such as immunization, vision and hearing care and oral care.

It is important to recognize that older people are heterogeneous, with varied health status and functional ability. Additionally, the social determinants of health greatly impact access to care and needs within communities. Diverse and tailored care and prevention is needed across the life course to support healthy ageing, accounting for social determinants, including and beyond age. Practitioners and program leaders should focus on developing practical guidance specific to their communities and local contexts and in recognition of the diversity of individuals as they age. While certain cities and communities may be more advanced in the implementation of age-friendly environments due to their socioeconomic status, globally health and social systems require reforms and community-embedded ecosystems to support healthy ageing and longevity. Capacity-building and resourcing is needed in low-and-middle income countries to strengthen health systems and age-friendliness, and address inequities between communities, countries and regions.

To advance policy and practice guidance, further research is needed to understand challenges and barriers for those working in age-friendly planning, collaboration across sectors to support integrating health service planning and age-friendly planning and tangible ways to apply best practice to other jurisdictions. Future research, with quantifiable and reproducible methodology, may focus on in depth analysis of health systems and practical approaches for age-friendly and health planning, particularly looking at enabling factors, such as health system organization, economic status and policies, in countries that have been successful in integrating health interventions within age-friendly plans. Additionally, future research is required to develop guidance for policy and practice change and long-term service operation and delivery at local levels.

## 7 Conclusion

An age-friendly future is one in which functional ability is maintained, equity is embedded in design, and public health is central to planning and delivery. As the United Nations Decade of Healthy Ageing reaches its midpoint, local governments, practitioners, and advocates must advance measurable, inclusive health interventions across all eight WHO domains. The examples highlighted in this study demonstrate a starting point for the prioritization of health interventions in age-friendly action plans. To ensure that no older person is left behind due to preventable or treatable health conditions, there is a need to embed public health in the core of age-friendly policy. The GNAFCC remains a critical platform for mutual learning and global progress.

## Data Availability

The original contributions presented in the study are included in the article/supplementary material, further inquiries can be directed to the corresponding author.
